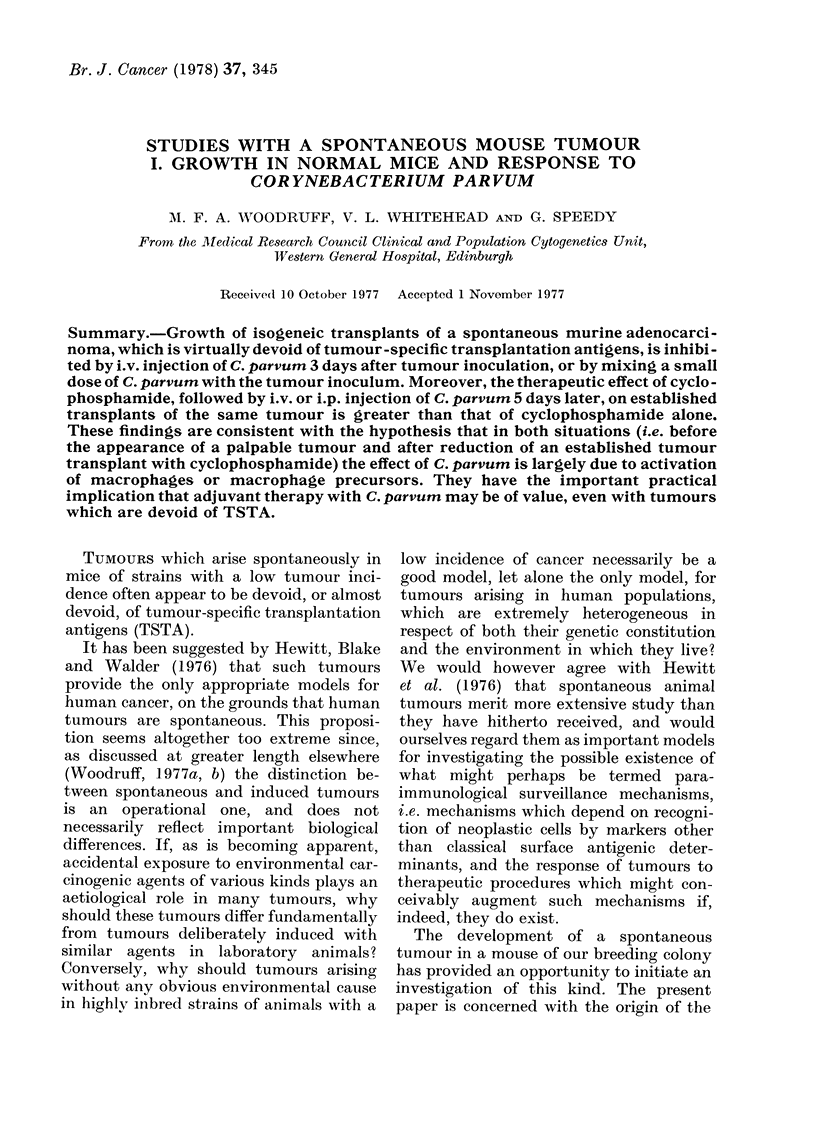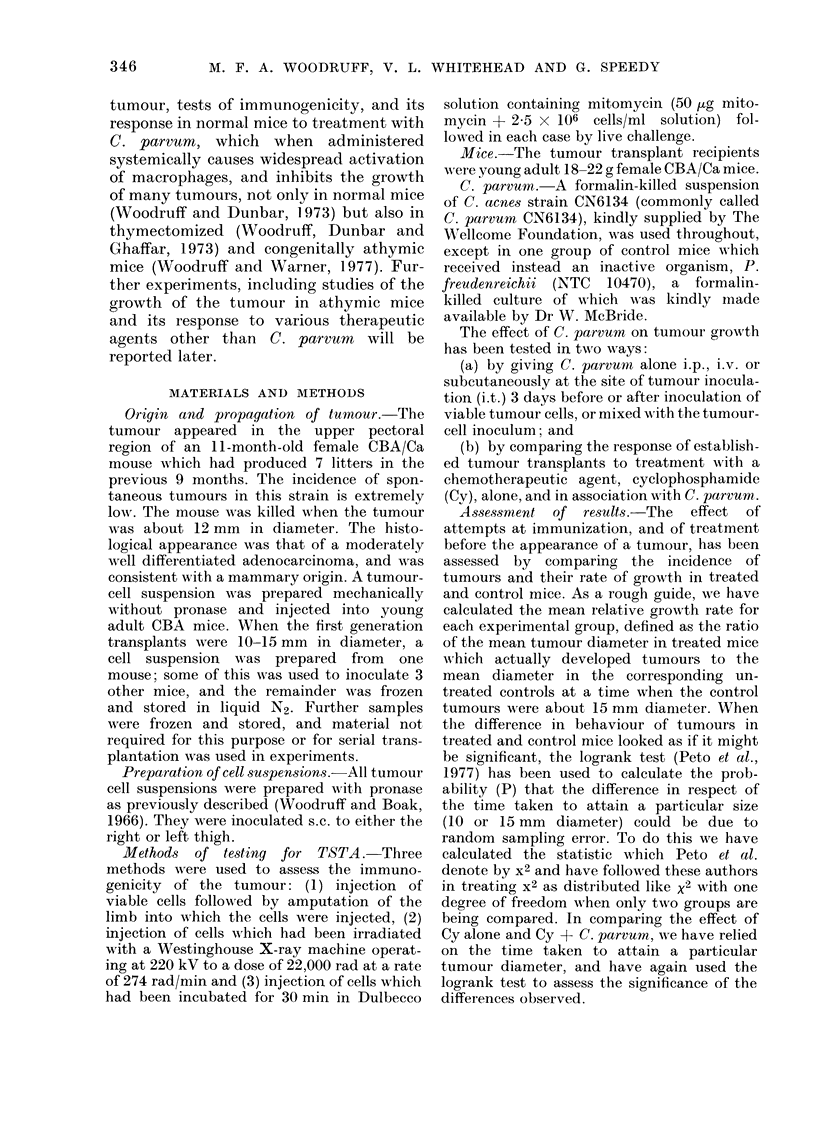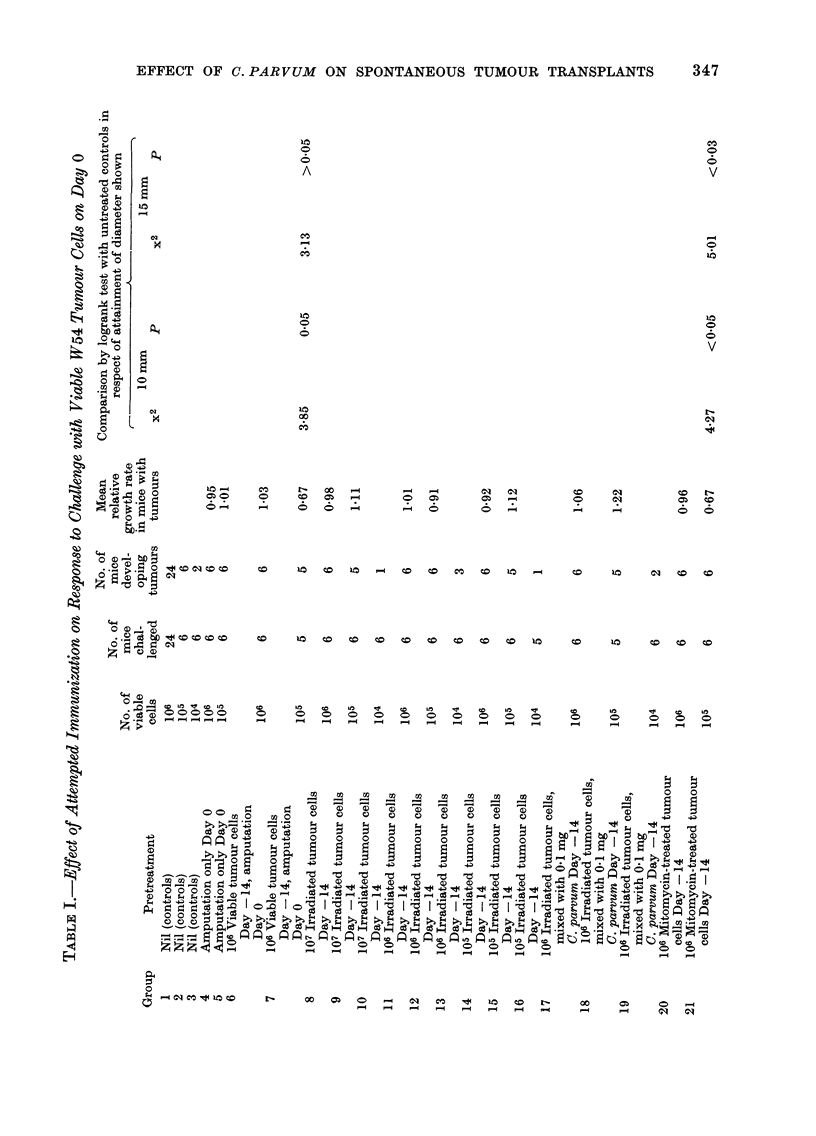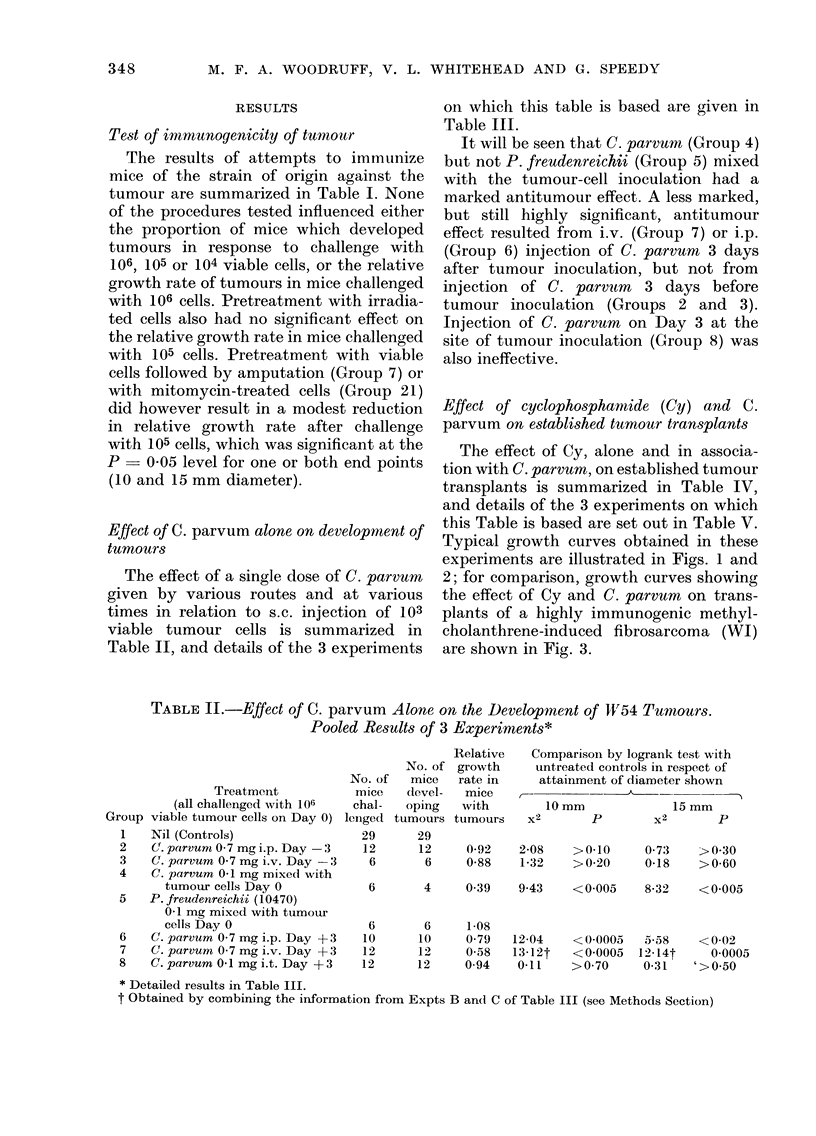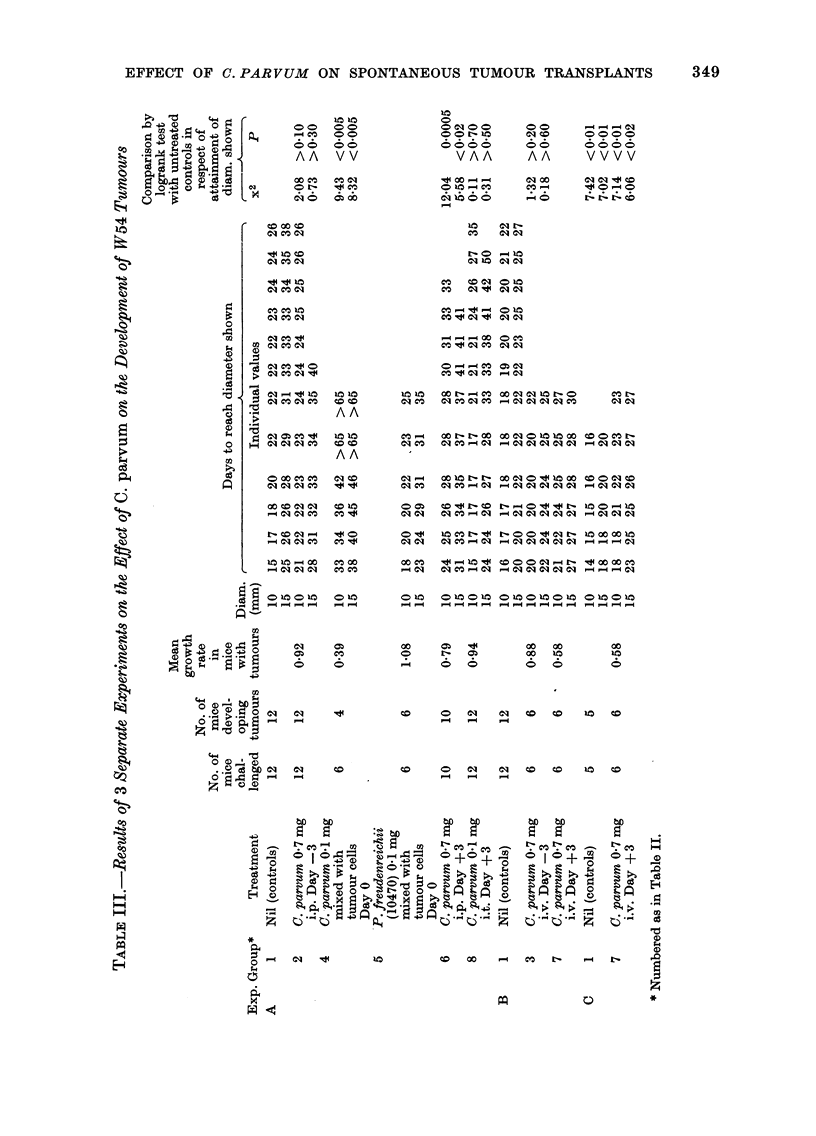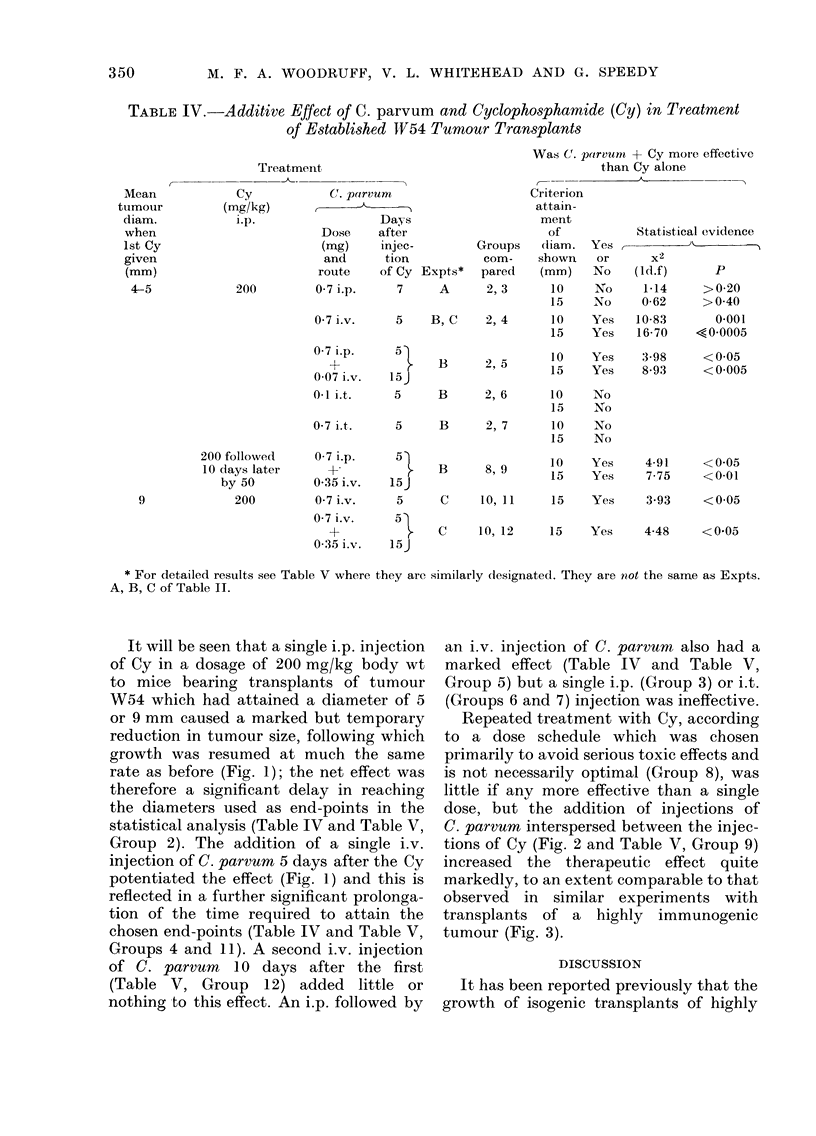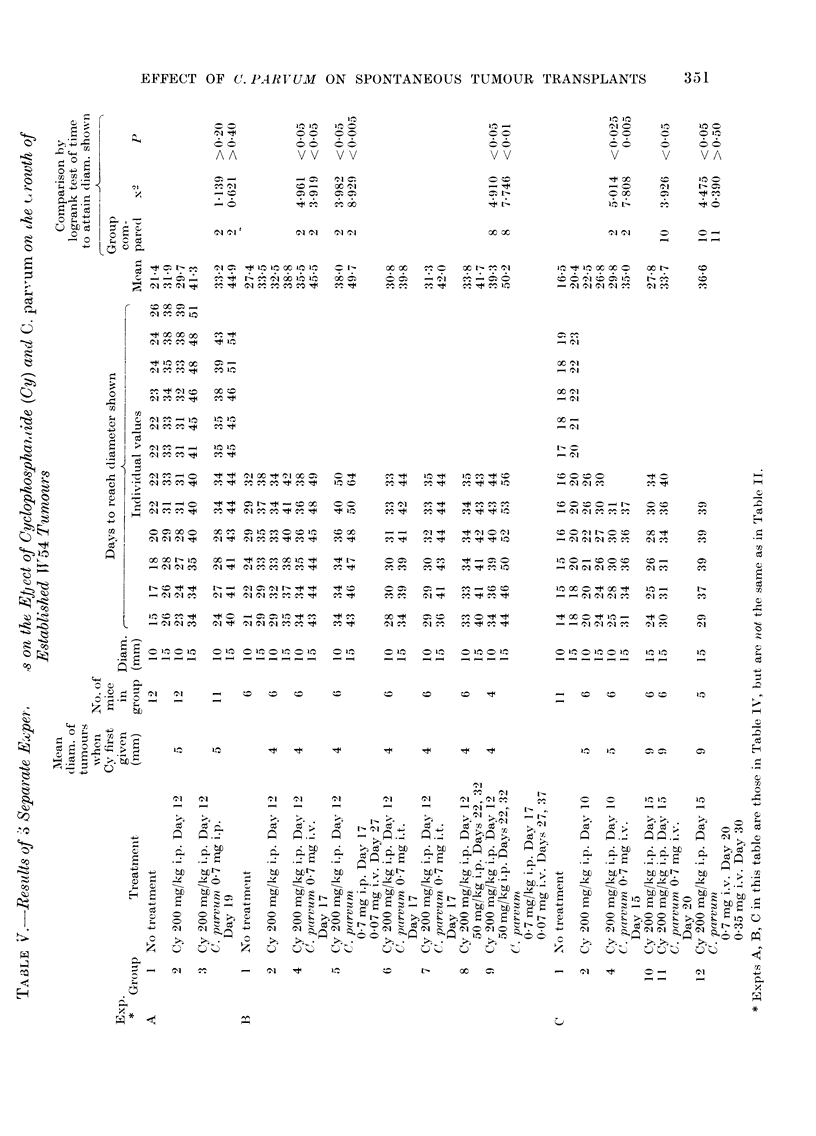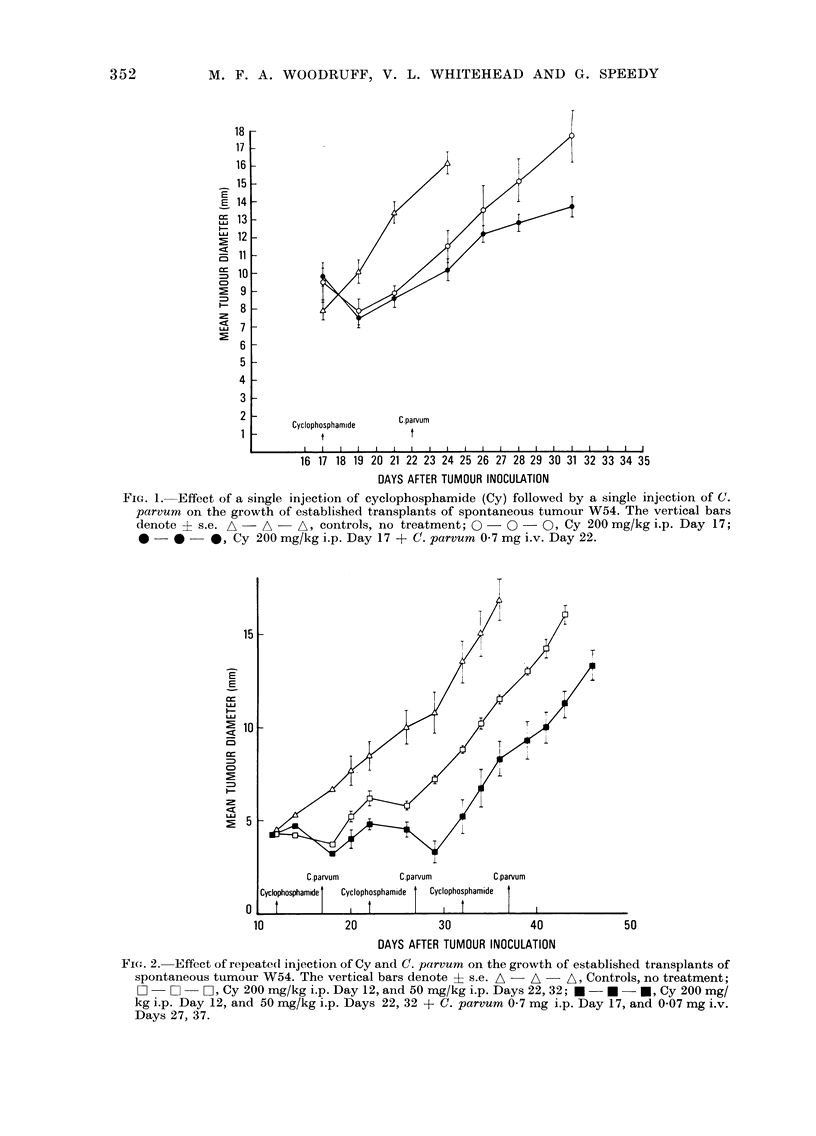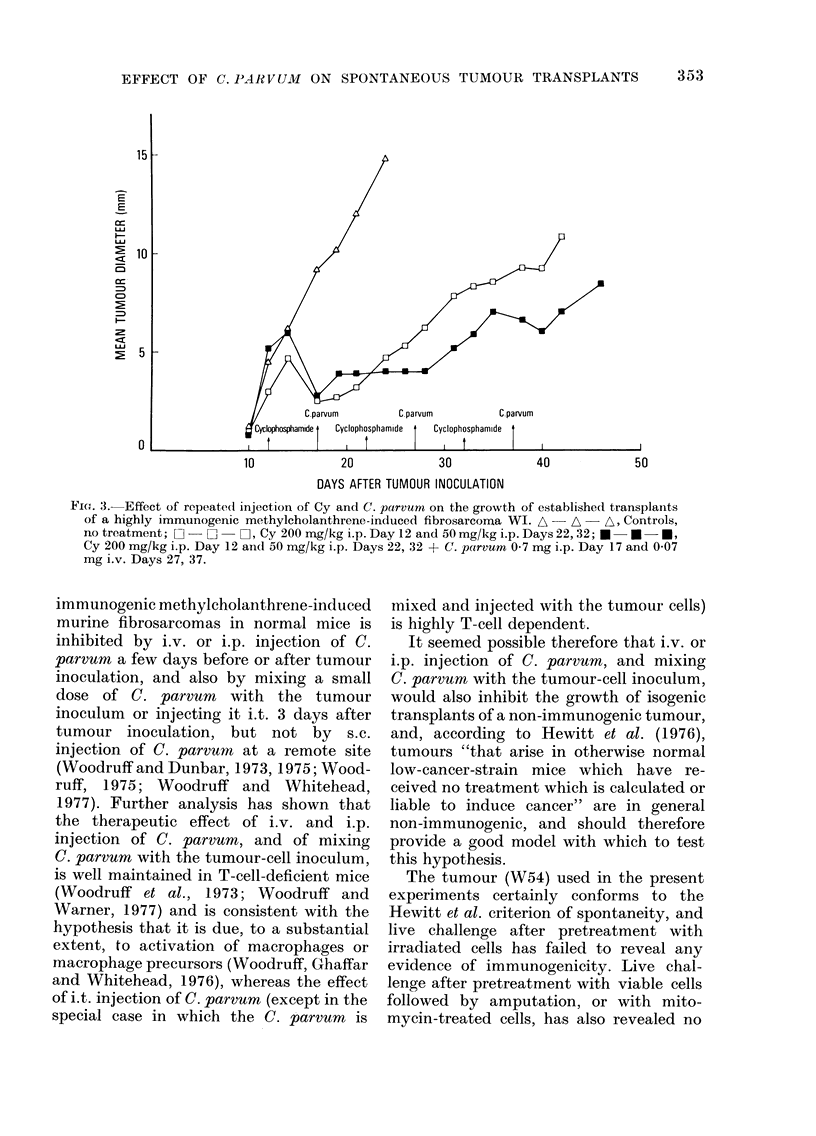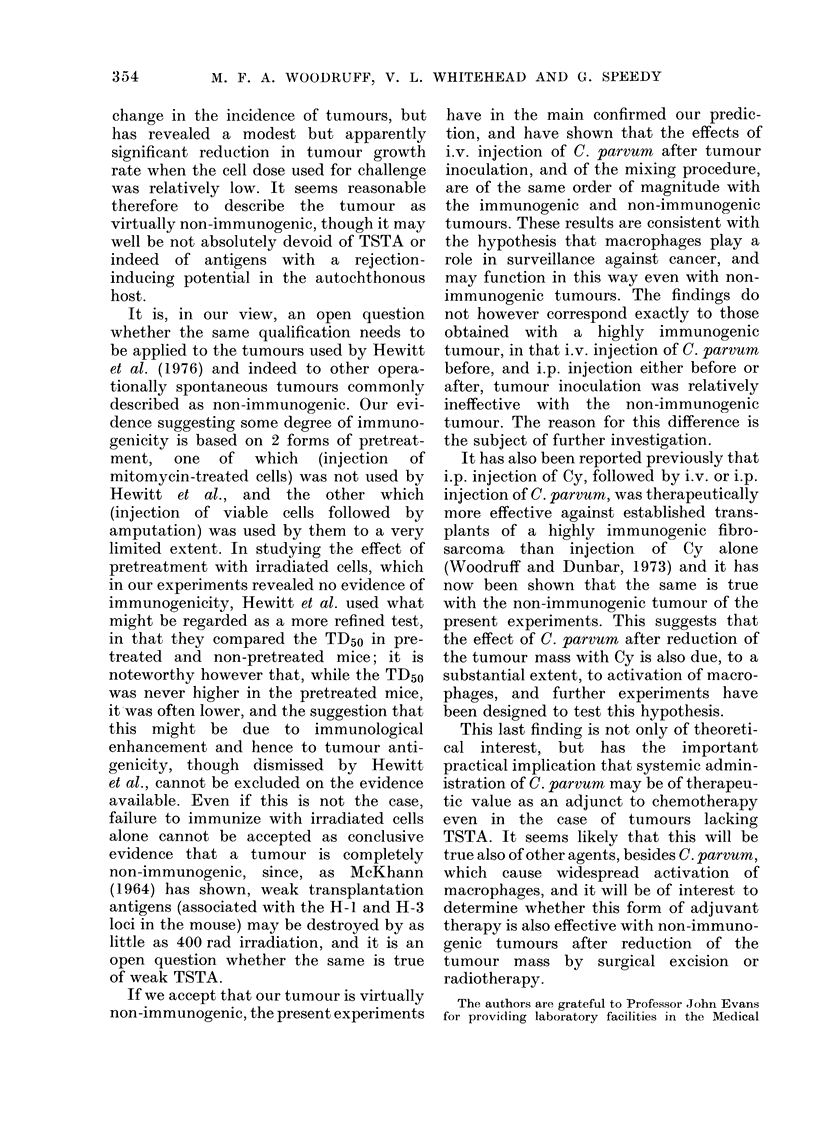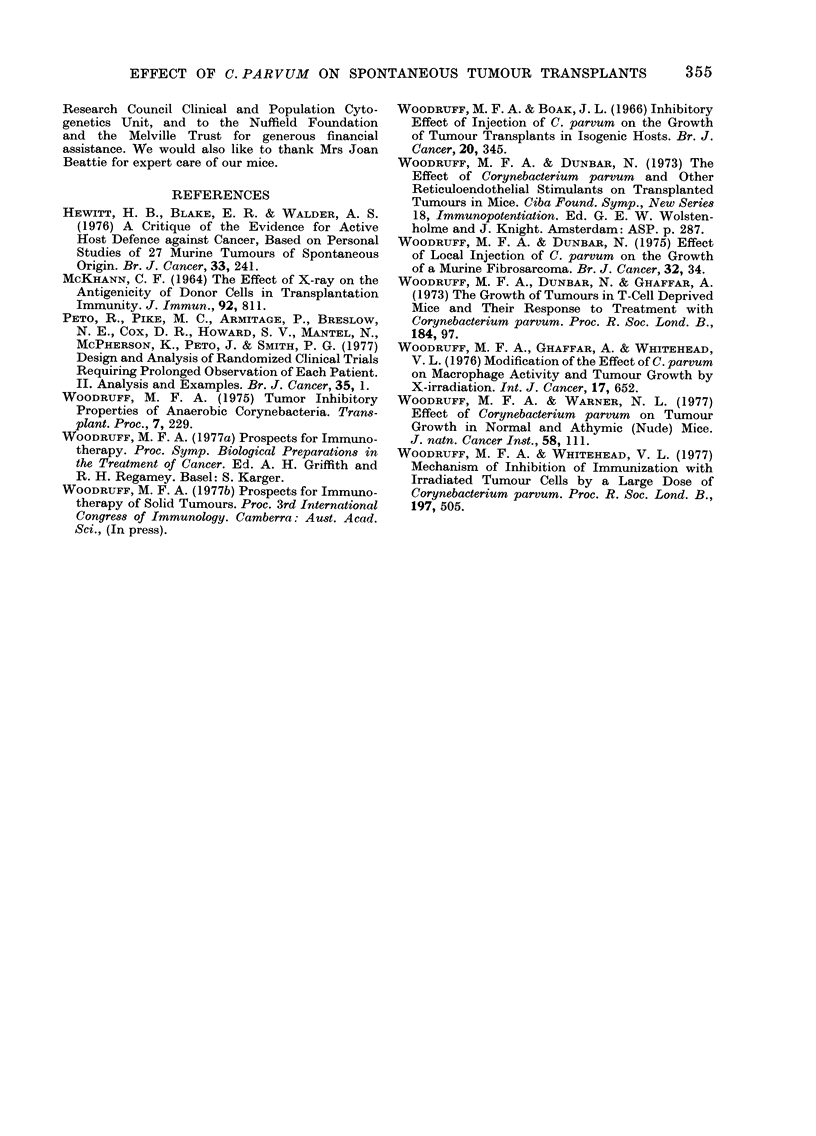# Studies with a spontaneous mouse tumor. I. Growth in normal mice and response to Corynebacterium parvum.

**DOI:** 10.1038/bjc.1978.53

**Published:** 1978-03

**Authors:** M. F. Woodruff, V. L. Whitehead, G. Speedy

## Abstract

Growth of isogeneic transplants of a spontaneous murine adenocarcinoma, which is virtually devoid of tumour-specific transplantation antigens, is inhibited by i.v. injection of C. parvum 3 days after tumour inoculation, or by mixing a small dose of C. parvum with the tumour inoculum. Moreover, the therapeutic effect of cyclophosphamide, followed by i.v. or i.p. injection of C. parvum 5 days later, on established transplants of the same tumour is greater than that of cyclophosphamide alone. These findings are consistent with the hypothesis that in both situations (i.e. before the appearance of a palpable tumour and after reduction of an established tumour transplant with cyclophosphamide) the effect of C. parvum is largely due to activation of macrophages or macrophage precursors. They have the important practical implication that adjuvant therapy with C. parvum may be of value, even with tumours which are devoid of TSTA.


*Br. J. Cancer* (1978) **37**, 345

### STUDIES WITH A SPONTANEOUS MOUSE TUMOUR I. GROWTH IN NORMAL MICE AND RESPONSE TO *CORYNEBACTERIUM PARVUM*

M. F. A. WOODRUFF, V. L. WHITEHEAD AND G. SPEEDY

*From the Medical Research Council Clinical and Population Cytogenetics Unit, Western General Hospital, Edinburgh*

Received 10 October 1977 Accepted 1 November 1977

**Summary.—Growth of isogeneic transplants of a spontaneous murine adenocarcinoma, which is virtually devoid of tumour-specific transplantation antigens, is inhibited by i.v. injection of *C. parvum* 3 days after tumour inoculation, or by mixing a small dose of *C. parvum* with the tumour inoculum. Moreover, the therapeutic effect of cyclophosphamide, followed by i.v. or i.p. injection of *C. parvum* 5 days later, on established transplants of the same tumour is greater than that of cyclophosphamide alone. These findings are consistent with the hypothesis that in both situations (*i.e.* before the appearance of a palpable tumour and after reduction of an established tumour transplant with cyclophosphamide) the effect of *C. parvum* is largely due to activation of macrophages or macrophage precursors. They have the important practical implication that adjuvant therapy with *C. parvum* may be of value, even with tumours which are devoid of TSTA.**

TUMOURS which arise spontaneously in mice of strains with a low tumour incidence often appear to be devoid, or almost devoid, of tumour-specific transplantation antigens (TSTA).

It has been suggested by Hewitt, Blake and Walder (1976) that such tumours provide the only appropriate models for human cancer, on the grounds that human tumours are spontaneous. This proposition seems altogether too extreme since, as discussed at greater length elsewhere (Woodruff, 1977*a*, *b*) the distinction between spontaneous and induced tumours is an operational one, and does not necessarily reflect important biological differences. If, as is becoming apparent, accidental exposure to environmental carcinogenic agents of various kinds plays an aetiological role in many tumours, why should these tumours differ fundamentally from tumours deliberately induced with similar agents in laboratory animals? Conversely, why should tumours arising without any obvious environmental cause in highly inbred strains of animals with a low incidence of cancer necessarily be a good model, let alone the only model, for tumours arising in human populations, which are extremely heterogeneous in respect of both their genetic constitution and the environment in which they live? We would however agree with Hewitt *et al.* (1976) that spontaneous animal tumours merit more extensive study than they have hitherto received, and would ourselves regard them as important models for investigating the possible existence of what might perhaps be termed para-immunological surveillance mechanisms, *i.e.* mechanisms which depend on recognition of neoplastic cells by markers other than classical surface antigenic determinants, and the response of tumours to therapeutic procedures which might conceivably augment such mechanisms if, indeed, they do exist.

The development of a spontaneous tumour in a mouse of our breeding colony has provided an opportunity to initiate an investigation of this kind. The present paper is concerned with the origin of the tumour, tests of immunogenicity, and its response in normal mice to treatment with *C. parvum*, which when administered systemically causes widespread activation of macrophages, and inhibits the growth of many tumours, not only in normal mice (Woodruff and Dunbar, 1973) but also in thymectomized (Woodruff, Dunbar and Ghaffar, 1973) and congenitally athymic mice (Woodruff and Warner, 1977). Further experiments, including studies of the growth of the tumour in athymic mice and its response to various therapeutic agents other than *C. parvum* will be reported later.

### MATERIALS AND METHODS

*Origin and propagation of tumour.*—The tumour appeared in the upper pectoral region of an 11-month-old female CBA/Ca mouse which had produced 7 litters in the previous 9 months. The incidence of spontaneous tumours in this strain is extremely low. The mouse was killed when the tumour was about 12 mm in diameter. The histological appearance was that of a moderately well differentiated adenocarcinoma, and was consistent with a mammary origin. A tumour-cell suspension was prepared mechanically without pronase and injected into young adult CBA mice. When the first generation transplants were 10–15 mm in diameter, a cell suspension was prepared from one mouse; some of this was used to inoculate 3 other mice, and the remainder was frozen and stored in liquid $N_2$. Further samples were frozen and stored, and material not required for this purpose or for serial transplantation was used in experiments.

*Preparation of cell suspensions.*—All tumour cell suspensions were prepared with pronase as previously described (Woodruff and Boak, 1966). They were inoculated s.c. to either the right or left thigh.

*Methods of testing for TSTA.*—Three methods were used to assess the immunogenicity of the tumour: (1) injection of viable cells followed by amputation of the limb into which the cells were injected, (2) injection of cells which had been irradiated with a Westinghouse **X**-ray machine operating at 220 kV to a dose of 22,000 rad at a rate of 274 rad/min and (3) injection of cells which had been incubated for 30 min in Dulbecco solution containing mitomycin (50 $\mu$g mitomycin $+ 2{\cdot}5 \times 10^6$ cells/ml solution) followed in each case by live challenge.

*Mice.*—The tumour transplant recipients were young adult 18–22 g female CBA/Ca mice.

*C. parvum.*—A formalin-killed suspension of *C. acnes* strain CN6134 (commonly called *C. parvum* CN6134), kindly supplied by The Wellcome Foundation, was used throughout, except in one group of control mice which received instead an inactive organism, *P. freudenreichii* (NTC 10470), a formalin-killed culture of which was kindly made available by Dr W. McBride.

The effect of *C. parvum* on tumour growth has been tested in two ways:

(a) by giving *C. parvum* alone i.p., i.v. or subcutaneously at the site of tumour inoculation (i.t.) 3 days before or after inoculation of viable tumour cells, or mixed with the tumour-cell inoculum; and

(b) by comparing the response of established tumour transplants to treatment with a chemotherapeutic agent, cyclophosphamide (Cy), alone, and in association with *C. parvum*.

*Assessment of results.*—The effect of attempts at immunization, and of treatment before the appearance of a tumour, has been assessed by comparing the incidence of tumours and their rate of growth in treated and control mice. As a rough guide, we have calculated the mean relative growth rate for each experimental group, defined as the ratio of the mean tumour diameter in treated mice which actually developed tumours to the mean diameter in the corresponding untreated controls at a time when the control tumours were about 15 mm diameter. When the difference in behaviour of tumours in treated and control mice looked as if it might be significant, the logrank test (Peto *et al.*, 1977) has been used to calculate the probability (P) that the difference in respect of the time taken to attain a particular size (10 or 15 mm diameter) could be due to random sampling error. To do this we have calculated the statistic which Peto *et al.* denote by $x^2$ and have followed these authors in treating $x^2$ as distributed like $\chi^2$ with one degree of freedom when only two groups are being compared. In comparing the effect of Cy alone and Cy + *C. parvum*, we have relied on the time taken to attain a particular tumour diameter, and have again used the logrank test to assess the significance of the differences observed.

TABLE I.—*Effect of Attempted Immunization on Response to Challenge with Viable W54 Tumour Cells on Day 0*

| Group | Pretreatment | No. of viable cells | No. of mice challenged | No. of mice developing tumours | Mean relative growth rate in mice with tumours | Comparison by logrank test with untreated controls in respect of attainment of diameter shown: 10 mm $x^2$ | 10 mm *P* | 15 mm $x^2$ | 15 mm *P* |
|---|---|---|---|---|---|---|---|---|---|
| 1 | Nil (controls) | $10^6$ | 24 | 24 | | | | | |
| 2 | Nil (controls) | $10^5$ | 6 | 6 | | | | | |
| 3 | Nil (controls) | $10^4$ | 6 | 2 | | | | | |
| 4 | Amputation only Day 0 | $10^6$ | 6 | 6 | 0·95 | | | | |
| 5 | Amputation only Day 0 | $10^5$ | 6 | 6 | 1·01 | | | | |
| 6 | $10^6$ Viable tumour cells Day −14, amputation Day 0 | $10^6$ | 6 | 6 | 1·03 | | | | |
| 7 | $10^6$ Viable tumour cells Day −14, amputation Day 0 | $10^5$ | 5 | 5 | 0·67 | 3·85 | 0·05 | 3·13 | >0·05 |
| 8 | $10^7$ Irradiated tumour cells Day −14 | $10^6$ | 6 | 6 | 0·98 | | | | |
| 9 | $10^7$ Irradiated tumour cells Day −14 | $10^5$ | 6 | 5 | 1·11 | | | | |
| 10 | $10^7$ Irradiated tumour cells Day −14 | $10^4$ | 6 | 1 | | | | | |
| 11 | $10^6$ Irradiated tumour cells Day −14 | $10^6$ | 6 | 6 | 1·01 | | | | |
| 12 | $10^6$ Irradiated tumour cells Day −14 | $10^5$ | 6 | 6 | 0·91 | | | | |
| 13 | $10^6$ Irradiated tumour cells Day −14 | $10^4$ | 6 | 3 | | | | | |
| 14 | $10^5$ Irradiated tumour cells Day −14 | $10^6$ | 6 | 6 | 0·92 | | | | |
| 15 | $10^5$ Irradiated tumour cells Day −14 | $10^5$ | 6 | 5 | 1·12 | | | | |
| 16 | $10^5$ Irradiated tumour cells Day −14 | $10^4$ | 5 | 1 | | | | | |
| 17 | $10^6$ Irradiated tumour cells, mixed with 0·1 mg *C. parvum* Day −14 | $10^6$ | 6 | 6 | 1·06 | | | | |
| 18 | $10^6$ Irradiated tumour cells, mixed with 0·1 mg *C. parvum* Day −14 | $10^5$ | 5 | 5 | 1·22 | | | | |
| 19 | $10^6$ Irradiated tumour cells, mixed with 0·1 mg *C. parvum* Day −14 | $10^4$ | 6 | 2 | | | | | |
| 20 | $10^6$ Mitomycin-treated tumour cells Day −14 | $10^6$ | 6 | 6 | 0·96 | | | | |
| 21 | $10^6$ Mitomycin-treated tumour cells Day −14 | $10^5$ | 6 | 6 | 0·67 | 4·27 | <0·05 | 5·01 | <0·03 |

### RESULTS

### *Test of immunogenicity of tumour*

The results of attempts to immunize mice of the strain of origin against the tumour are summarized in Table I. None of the procedures tested influenced either the proportion of mice which developed tumours in response to challenge with $10^6$, $10^5$ or $10^4$ viable cells, or the relative growth rate of tumours in mice challenged with $10^6$ cells. Pretreatment with irradiated cells also had no significant effect on the relative growth rate in mice challenged with $10^5$ cells. Pretreatment with viable cells followed by amputation (Group 7) or with mitomycin-treated cells (Group 21) did however result in a modest reduction in relative growth rate after challenge with $10^5$ cells, which was significant at the $P = 0{\cdot}05$ level for one or both end points (10 and 15 mm diameter).

### *Effect of* C. parvum *alone on development of tumours*

The effect of a single dose of *C. parvum* given by various routes and at various times in relation to s.c. injection of $10^3$ viable tumour cells is summarized in Table II, and details of the 3 experiments on which this table is based are given in Table III.

It will be seen that *C. parvum* (Group 4) but not *P. freudenreichii* (Group 5) mixed with the tumour-cell inoculation had a marked antitumour effect. A less marked, but still highly significant, antitumour effect resulted from i.v. (Group 7) or i.p. (Group 6) injection of *C. parvum* 3 days after tumour inoculation, but not from injection of *C. parvum* 3 days before tumour inoculation (Groups 2 and 3). Injection of *C. parvum* on Day 3 at the site of tumour inoculation (Group 8) was also ineffective.

### *Effect of cyclophosphamide (Cy) and* C. parvum *on established tumour transplants*

The effect of Cy, alone and in association with *C. parvum*, on established tumour transplants is summarized in Table IV, and details of the 3 experiments on which this Table is based are set out in Table V. Typical growth curves obtained in these experiments are illustrated in Figs. 1 and 2; for comparison, growth curves showing the effect of Cy and *C. parvum* on transplants of a highly immunogenic methylcholanthrene-induced fibrosarcoma (WI) are shown in Fig. 3.

TABLE II.—*Effect of* C. parvum *Alone on the Development of W54 Tumours. Pooled Results of 3 Experiments**

| Group | Treatment (all challenged with $10^6$ viable tumour cells on Day 0) | No. of mice challenged | No. of mice developing tumours | Relative growth rate in mice with tumours | Comparison by logrank test with untreated controls in respect of attainment of diameter shown: 10 mm $x^2$ | 10 mm $P$ | 15 mm $x^2$ | 15 mm $P$ |
|---|---|---|---|---|---|---|---|---|
| 1 | Nil (Controls) | 29 | 29 | | | | | |
| 2 | *C. parvum* 0·7 mg i.p. Day −3 | 12 | 12 | 0·92 | 2·08 | >0·10 | 0·73 | >0·30 |
| 3 | *C. parvum* 0·7 mg i.v. Day −3 | 6 | 6 | 0·88 | 1·32 | >0·20 | 0·18 | >0·60 |
| 4 | *C. parvum* 0·1 mg mixed with tumour cells Day 0 | 6 | 4 | 0·39 | 9·43 | <0·005 | 8·32 | <0·005 |
| 5 | *P. freudenreichii* (10470) 0·1 mg mixed with tumour cells Day 0 | 6 | 6 | 1·08 | | | | |
| 6 | *C. parvum* 0·7 mg i.p. Day +3 | 10 | 10 | 0·79 | 12·04 | <0·0005 | 5·58 | <0·02 |
| 7 | *C. parvum* 0·7 mg i.v. Day +3 | 12 | 12 | 0·58 | 13·12† | <0·0005 | 12·14† | 0·0005 |
| 8 | *C. parvum* 0·1 mg i.t. Day +3 | 12 | 12 | 0·94 | 0·11 | >0·70 | 0·31 | '>0·50 |

* Detailed results in Table III.

† Obtained by combining the information from Expts B and C of Table III (see Methods Section)

TABLE III.—*Results of 3 Separate Experiments on the Effect of* C. parvum *on the Development of W54 Tumours*

| Exp. | Group* | Treatment | No. of mice challenged | No. of mice developing tumours | Mean growth rate in mice with tumours | Diam. (mm) | Days to reach diameter shown: Individual values | | | | | | | | | | | | Comparison by logrank test with untreated controls in respect of attainment of diam. shown: $x^2$ | P |
|---|---|---|---|---|---|---|---|---|---|---|---|---|---|---|---|---|---|---|---|---|
| A | 1 | Nil (controls) | 12 | 12 | | 10 | 15 | 17 | 18 | 20 | 22 | 22 | 22 | 22 | 23 | 24 | 24 | 26 | | |
| | | | | | | 15 | 25 | 26 | 26 | 28 | 29 | 31 | 33 | 33 | 33 | 34 | 35 | 38 | | |
| | 2 | *C. parvum* 0·7 mg | 12 | 12 | 0·92 | 10 | 21 | 22 | 22 | 23 | 23 | 24 | 24 | 24 | 25 | 25 | 26 | 26 | 2·08 | >0·10 |
| | | i.p. Day −3 | | | | 15 | 28 | 31 | 32 | 33 | 34 | 35 | 40 | | | | | | 0·73 | >0·30 |
| | 4 | *C. parvum* 0·1 mg mixed with | 6 | 4 | 0·39 | 10 | 33 | 34 | 36 | 42 | >65 | >65 | | | | | | | 9·43 | <0·005 |
| | | tumour cells Day 0 | | | | 15 | 38 | 40 | 45 | 46 | >65 | >65 | | | | | | | 8·32 | <0·005 |
| | 5 | *P. freudenreichii* (10470) 0·1 mg mixed with | 6 | 6 | 1·08 | 10 | 18 | 20 | 20 | 22 | 23 | 25 | | | | | | | | |
| | | tumour cells Day 0 | | | | 15 | 23 | 24 | 29 | 31 | 31 | 35 | | | | | | | | |
| | 6 | *C. parvum* 0·7 mg | 10 | 10 | 0·79 | 10 | 24 | 25 | 26 | 28 | 28 | 28 | 30 | 31 | 33 | 33 | | | 12·04 | 0·0005 |
| | | i.p. Day +3 | | | | 15 | 31 | 33 | 34 | 35 | 37 | 37 | 41 | 41 | 41 | | | | 5·58 | <0·02 |
| | 8 | *C. parvum* 0·1 mg | 12 | 12 | 0·94 | 10 | 15 | 17 | 17 | 17 | 17 | 21 | 21 | 21 | 24 | 26 | 27 | 35 | 0·11 | >0·70 |
| | | i.t. Day +3 | | | | 15 | 24 | 24 | 26 | 27 | 28 | 33 | 33 | 38 | 41 | 42 | 50 | | 0·31 | >0·50 |
| B | 1 | Nil (controls) | 12 | 12 | | 10 | 16 | 17 | 17 | 18 | 18 | 18 | 19 | 20 | 20 | 20 | 21 | 22 | | |
| | | | | | | 15 | 20 | 20 | 21 | 22 | 22 | 22 | 22 | 23 | 25 | 25 | 25 | 27 | | |
| | 3 | *C. parvum* 0·7 mg | 6 | 6 | 0·88 | 10 | 20 | 20 | 20 | 20 | 20 | 22 | | | | | | | 1·32 | >0·20 |
| | | i.v. Day −3 | | | | 15 | 22 | 24 | 24 | 24 | 25 | 25 | | | | | | | 0·18 | >0·60 |
| | 7 | *C. parvum* 0·7 mg | 6 | 6 | 0·58 | 10 | 21 | 22 | 24 | 25 | 25 | 27 | | | | | | | | |
| | | i.v. Day +3 | | | | 15 | 27 | 27 | 27 | 28 | 28 | 30 | | | | | | | | |
| C | 1 | Nil (controls) | 5 | 5 | | 10 | 14 | 15 | 15 | 16 | 16 | | | | | | | | 7·42 | <0·01 |
| | | | | | | 15 | 18 | 18 | 20 | 20 | 20 | | | | | | | | 7·02 | <0·01 |
| | 7 | *C. parvum* 0·7 mg | 6 | 6 | 0·58 | 10 | 18 | 18 | 21 | 22 | 23 | 23 | | | | | | | 7·14 | <0·01 |
| | | i.v. Day +3 | | | | 15 | 23 | 25 | 25 | 26 | 27 | 27 | | | | | | | 6·06 | <0·02 |

* Numbered as in Table II.

TABLE IV.—*Additive Effect of* C. parvum *and Cyclophosphamide* (*Cy*) *in Treatment of Established W54 Tumour Transplants*

| Treatment | | | | | | Was *C. parvum* + Cy more effective than Cy alone | | | |
|---|---|---|---|---|---|---|---|---|---|
| Mean tumour diam. when 1st Cy given (mm) | Cy (mg/kg) i.p. | *C. parvum* Dose (mg) and route | *C. parvum* Days after injection of Cy | Expts* | Groups compared | Criterion attainment of diam. shown (mm) | Yes or No | Statistical evidence $\chi^2$ (1d.f) | Statistical evidence *P* |
| 4–5 | 200 | 0·7 i.p. | 7 | A | 2, 3 | 10 | No | 1·14 | >0·20 |
| | | | | | | 15 | No | 0·62 | >0·40 |
| | | 0·7 i.v. | 5 | B, C | 2, 4 | 10 | Yes | 10·83 | 0·001 |
| | | | | | | 15 | Yes | 16·70 | ≤0·0005 |
| | | 0·7 i.p. + 0·07 i.v. | 5 } 15 } | B | 2, 5 | 10 | Yes | 3·98 | <0·05 |
| | | | | | | 15 | Yes | 8·93 | <0·005 |
| | | 0·1 i.t. | 5 | B | 2, 6 | 10 | No | | |
| | | | | | | 15 | No | | |
| | | 0·7 i.t. | 5 | B | 2, 7 | 10 | No | | |
| | | | | | | 15 | No | | |
| | 200 followed 10 days later by 50 | 0·7 i.p. + 0·35 i.v. | 5 } 15 } | B | 8, 9 | 10 | Yes | 4·91 | <0·05 |
| | | | | | | 15 | Yes | 7·75 | <0·01 |
| 9 | 200 | 0·7 i.v. | 5 | C | 10, 11 | 15 | Yes | 3·93 | <0·05 |
| | | 0·7 i.v. + 0·35 i.v. | 5 } 15 } | C | 10, 12 | 15 | Yes | 4·48 | <0·05 |

* For detailed results see Table V where they are similarly designated. They are *not* the same as Expts. A, B, C of Table II.

It will be seen that a single i.p. injection of Cy in a dosage of 200 mg/kg body wt to mice bearing transplants of tumour W54 which had attained a diameter of 5 or 9 mm caused a marked but temporary reduction in tumour size, following which growth was resumed at much the same rate as before (Fig. 1); the net effect was therefore a significant delay in reaching the diameters used as end-points in the statistical analysis (Table IV and Table V, Group 2). The addition of a single i.v. injection of *C. parvum* 5 days after the Cy potentiated the effect (Fig. 1) and this is reflected in a further significant prolongation of the time required to attain the chosen end-points (Table IV and Table V, Groups 4 and 11). A second i.v. injection of *C. parvum* 10 days after the first (Table V, Group 12) added little or nothing to this effect. An i.p. followed by an i.v. injection of *C. parvum* also had a marked effect (Table IV and Table V, Group 5) but a single i.p. (Group 3) or i.t. (Groups 6 and 7) injection was ineffective.

Repeated treatment with Cy, according to a dose schedule which was chosen primarily to avoid serious toxic effects and is not necessarily optimal (Group 8), was little if any more effective than a single dose, but the addition of injections of *C. parvum* interspersed between the injections of Cy (Fig. 2 and Table V, Group 9) increased the therapeutic effect quite markedly, to an extent comparable to that observed in similar experiments with transplants of a highly immunogenic tumour (Fig. 3).

### DISCUSSION

It has been reported previously that the growth of isogenic transplants of highly

TABLE V.—*Results of 3 Separate Exper*[illegible]*s on the Effect of Cyclophospha*[illegible]*ide (Cy) and* C. parvum *on the* [illegible]*rowth of Established W54 Tumours*

| Exp.* | Group | Treatment | Mean diam. of tumours when Cy first given (mm) | No. of mice in group | Diam. (mm) | Days to reach diameter shown: Individual values | | | | | | | | | | | | Mean | Comparison by logrank test of time to attain diam. shown: Group compared | $x^2$ | *P* |
|---|---|---|---|---|---|---|---|---|---|---|---|---|---|---|---|---|---|---|---|---|---|
| A | 1 | No treatment | | 12 | 10 | 15 | 17 | 18 | 20 | 22 | 22 | 22 | 22 | 23 | 24 | 24 | 26 | 21·4 | | | |
| | | | | | 15 | 26 | 26 | 28 | 29 | 31 | 33 | 33 | 33 | 34 | 35 | 38 | 38 | 31·9 | | | |
| | 2 | Cy 200 mg/kg i.p. Day 12 | 5 | 12 | 10 | 23 | 24 | 27 | 28 | 31 | 31 | 31 | 31 | 32 | 33 | 38 | 39 | 29·7 | | | |
| | | | | | 15 | 34 | 34 | 35 | 40 | 40 | 40 | 41 | 45 | 46 | 48 | 48 | 51 | 41·3 | | | |
| | 3 | Cy 200 mg/kg i.p. Day 12 *C. parvum* 0·7 mg i.p. | 5 | 11 | 10 | 24 | 27 | 28 | 28 | 34 | 34 | 35 | 35 | 38 | 39 | 43 | | 33·2 | 2 | 1·139 | >0·20 |
| | | Day 19 | | | 15 | 40 | 41 | 41 | 43 | 44 | 44 | 45 | 45 | 46 | 51 | 54 | | 44·9 | 2 | 0·621 | >0·40 |
| B | 1 | No treatment | | 6 | 10 | 21 | 22 | 24 | 29 | 29 | 32 | | | | | | | 27·4 | | | |
| | | | | | 15 | 29 | 29 | 33 | 35 | 37 | 38 | | | | | | | 33·5 | | | |
| | 2 | Cy 200 mg/kg i.p. Day 12 | 4 | 6 | 10 | 29 | 32 | 33 | 33 | 34 | 34 | | | | | | | 32·5 | | | |
| | | | | | 15 | 35 | 37 | 38 | 40 | 41 | 42 | | | | | | | 38·8 | | | |
| | 4 | Cy 200 mg/kg i.p. Day 12 | 4 | 6 | 10 | 34 | 34 | 35 | 36 | 36 | 38 | | | | | | | 35·5 | 2 | 4·961 | <0·05 |
| | | *C. parvum* 0·7 mg i.v. Day 17 | | | 15 | 43 | 44 | 44 | 45 | 48 | 49 | | | | | | | 45·5 | 2 | 3·919 | <0·05 |
| | 5 | Cy 200 mg/kg i.p. Day 12 | 4 | 6 | 10 | 34 | 34 | 34 | 36 | 40 | 50 | | | | | | | 38·0 | 2 | 3·982 | <0·05 |
| | | *C. parvum* 0·7 mg i.p. Day 17 0·07 mg i.v. Day 27 | | | 15 | 43 | 46 | 47 | 48 | 50 | 64 | | | | | | | 49·7 | 2 | 8·929 | <0·005 |
| | 6 | Cy 200 mg/kg i.p. Day 12 | 4 | 6 | 10 | 28 | 30 | 30 | 31 | 33 | 33 | | | | | | | 30·8 | | | |
| | | *C. parvum* 0·7 mg i.t. Day 17 | | | 15 | 34 | 39 | 39 | 41 | 42 | 44 | | | | | | | 39·8 | | | |
| | 7 | Cy 200 mg/kg i.p. Day 12 | 4 | 6 | 10 | 29 | 29 | 30 | 32 | 33 | 35 | | | | | | | 31·3 | | | |
| | | *C. parvum* 0·7 mg i.t. Day 17 | | | 15 | 36 | 41 | 43 | 44 | 44 | 44 | | | | | | | 42·0 | | | |
| | 8 | Cy 200 mg/kg i.p. Day 12 | 4 | 6 | 10 | 33 | 33 | 34 | 34 | 34 | 35 | | | | | | | 33·8 | | | |
| | | 50 mg/kg i.p. Days 22, 32 | | | 15 | 40 | 41 | 41 | 42 | 43 | 43 | | | | | | | 41·7 | | | |
| | 9 | Cy 200 mg/kg i.p. Day 12 | 4 | 4 | 10 | 34 | 36 | 39 | 40 | 43 | 44 | | | | | | | 39·3 | 8 | 4·910 | <0·05 |
| | | 50 mg/kg i.p. Days 22, 32 *C. parvum* 0·7 mg/kg i.p. Day 17 0·07 mg i.v. Days 27, 37 | | | 15 | 44 | 46 | 50 | 52 | 53 | 56 | | | | | | | 50·2 | 8 | 7·746 | <0·01 |
| C | 1 | No treatment | | 11 | 10 | 14 | 15 | 15 | 16 | 16 | 16 | 17 | 18 | 18 | 18 | 19 | | 16·5 | | | |
| | | | | | 15 | 18 | 18 | 20 | 20 | 20 | 20 | 20 | 21 | 22 | 22 | 23 | | 20·4 | | | |
| | 2 | Cy 200 mg/kg i.p. Day 10 | 5 | 6 | 10 | 20 | 20 | 21 | 22 | 26 | 26 | | | | | | | 22·5 | | | |
| | | | | | 15 | 24 | 24 | 26 | 27 | 30 | 30 | | | | | | | 26·8 | | | |
| | 4 | Cy 200 mg/kg i.p. Day 10 | 5 | 6 | 10 | 25 | 28 | 30 | 30 | 31 | | | | | | | | 29·8 | 2 | 5·014 | <0·025 |
| | | *C. parvum* 0·7 mg i.v. Day 15 | | | 15 | 31 | 34 | 36 | 36 | 37 | | | | | | | | 35·0 | 2 | 7·808 | 0·005 |
| | 10 | Cy 200 mg/kg i.p. Day 15 | 9 | 6 | 15 | 24 | 25 | 26 | 28 | 30 | 34 | | | | | | | 27·8 | | | |
| | 11 | Cy 200 mg/kg i.p. Day 15 *C. parvum* 0·7 mg i.v. Day 20 | 9 | 6 | 15 | 30 | 31 | 31 | 34 | 36 | 40 | | | | | | | 33·7 | 10 | 3·926 | <0·05 |
| | 12 | Cy 200 mg/kg i.p. Day 15 | 9 | 5 | 15 | 29 | 37 | 39 | 39 | 39 | | | | | | | | 36·6 | 10 | 4·475 | <0·05 |
| | | *C. parvum* 0·7 mg i.v. Day 20 0·35 mg i.v. Day 30 | | | | | | | | | | | | | | | | | 11 | 0·390 | >0·50 |

* Expts A, B, C in this table are those in Table IV, but are *not* the same as in Table II.

FIG. 1.—Effect of a single injection of cyclophosphamide (Cy) followed by a single injection of *C. parvum* on the growth of established transplants of spontaneous tumour W54. The vertical bars denote ± s.e. △ — △ — △, controls, no treatment; ○ — ○ — ○, Cy 200 mg/kg i.p. Day 17; ● — ● — ●, Cy 200 mg/kg i.p. Day 17 + *C. parvum* 0·7 mg i.v. Day 22.

FIG. 2.—Effect of repeated injection of Cy and *C. parvum* on the growth of established transplants of spontaneous tumour W54. The vertical bars denote ± s.e. △ — △ — △, Controls, no treatment; □ — □ — □, Cy 200 mg/kg i.p. Day 12, and 50 mg/kg i.p. Days 22, 32; ■ — ■ — ■, Cy 200 mg/kg i.p. Day 12, and 50 mg/kg i.p. Days 22, 32 + *C. parvum* 0·7 mg i.p. Day 17, and 0·07 mg i.v. Days 27, 37.

FIG. 3.—Effect of repeated injection of Cy and *C. parvum* on the growth of established transplants of a highly immunogenic methylcholanthrene-induced fibrosarcoma WI. △ — △ — △, Controls, no treatment; □ — □ — □, Cy 200 mg/kg i.p. Day 12 and 50 mg/kg i.p. Days 22, 32; ■ — ■ — ■, Cy 200 mg/kg i.p. Day 12 and 50 mg/kg i.p. Days 22, 32 + *C. parvum* 0·7 mg i.p. Day 17 and 0·07 mg i.v. Days 27, 37.

immunogenic methylcholanthrene-induced murine fibrosarcomas in normal mice is inhibited by i.v. or i.p. injection of *C. parvum* a few days before or after tumour inoculation, and also by mixing a small dose of *C. parvum* with the tumour inoculum or injecting it i.t. 3 days after tumour inoculation, but not by s.c. injection of *C. parvum* at a remote site (Woodruff and Dunbar, 1973, 1975; Woodruff, 1975; Woodruff and Whitehead, 1977). Further analysis has shown that the therapeutic effect of i.v. and i.p. injection of *C. parvum*, and of mixing *C. parvum* with the tumour-cell inoculum, is well maintained in T-cell-deficient mice (Woodruff *et al.*, 1973; Woodruff and Warner, 1977) and is consistent with the hypothesis that it is due, to a substantial extent, to activation of macrophages or macrophage precursors (Woodruff, Ghaffar and Whitehead, 1976), whereas the effect of i.t. injection of *C. parvum* (except in the special case in which the *C. parvum* is mixed and injected with the tumour cells) is highly T-cell dependent.

It seemed possible therefore that i.v. or i.p. injection of *C. parvum*, and mixing *C. parvum* with the tumour-cell inoculum, would also inhibit the growth of isogenic transplants of a non-immunogenic tumour, and, according to Hewitt *et al.* (1976), tumours "that arise in otherwise normal low-cancer-strain mice which have received no treatment which is calculated or liable to induce cancer" are in general non-immunogenic, and should therefore provide a good model with which to test this hypothesis.

The tumour (W54) used in the present experiments certainly conforms to the Hewitt *et al.* criterion of spontaneity, and live challenge after pretreatment with irradiated cells has failed to reveal any evidence of immunogenicity. Live challenge after pretreatment with viable cells followed by amputation, or with mitomycin-treated cells, has also revealed no change in the incidence of tumours, but has revealed a modest but apparently significant reduction in tumour growth rate when the cell dose used for challenge was relatively low. It seems reasonable therefore to describe the tumour as virtually non-immunogenic, though it may well be not absolutely devoid of TSTA or indeed of antigens with a rejection-inducing potential in the autochthonous host.

It is, in our view, an open question whether the same qualification needs to be applied to the tumours used by Hewitt *et al.* (1976) and indeed to other operationally spontaneous tumours commonly described as non-immunogenic. Our evidence suggesting some degree of immunogenicity is based on 2 forms of pretreatment, one of which (injection of mitomycin-treated cells) was not used by Hewitt *et al.*, and the other which (injection of viable cells followed by amputation) was used by them to a very limited extent. In studying the effect of pretreatment with irradiated cells, which in our experiments revealed no evidence of immunogenicity, Hewitt *et al.* used what might be regarded as a more refined test, in that they compared the $TD_{50}$ in pretreated and non-pretreated mice; it is noteworthy however that, while the $TD_{50}$ was never higher in the pretreated mice, it was often lower, and the suggestion that this might be due to immunological enhancement and hence to tumour antigenicity, though dismissed by Hewitt *et al.*, cannot be excluded on the evidence available. Even if this is not the case, failure to immunize with irradiated cells alone cannot be accepted as conclusive evidence that a tumour is completely non-immunogenic, since, as McKhann (1964) has shown, weak transplantation antigens (associated with the H-1 and H-3 loci in the mouse) may be destroyed by as little as 400 rad irradiation, and it is an open question whether the same is true of weak TSTA.

If we accept that our tumour is virtually non-immunogenic, the present experiments have in the main confirmed our prediction, and have shown that the effects of i.v. injection of *C. parvum* after tumour inoculation, and of the mixing procedure, are of the same order of magnitude with the immunogenic and non-immunogenic tumours. These results are consistent with the hypothesis that macrophages play a role in surveillance against cancer, and may function in this way even with non-immunogenic tumours. The findings do not however correspond exactly to those obtained with a highly immunogenic tumour, in that i.v. injection of *C. parvum* before, and i.p. injection either before or after, tumour inoculation was relatively ineffective with the non-immunogenic tumour. The reason for this difference is the subject of further investigation.

It has also been reported previously that i.p. injection of Cy, followed by i.v. or i.p. injection of *C. parvum*, was therapeutically more effective against established transplants of a highly immunogenic fibrosarcoma than injection of Cy alone (Woodruff and Dunbar, 1973) and it has now been shown that the same is true with the non-immunogenic tumour of the present experiments. This suggests that the effect of *C. parvum* after reduction of the tumour mass with Cy is also due, to a substantial extent, to activation of macrophages, and further experiments have been designed to test this hypothesis.

This last finding is not only of theoretical interest, but has the important practical implication that systemic administration of *C. parvum* may be of therapeutic value as an adjunct to chemotherapy even in the case of tumours lacking TSTA. It seems likely that this will be true also of other agents, besides *C. parvum*, which cause widespread activation of macrophages, and it will be of interest to determine whether this form of adjuvant therapy is also effective with non-immunogenic tumours after reduction of the tumour mass by surgical excision or radiotherapy.

The authors are grateful to Professor John Evans for providing laboratory facilities in the Medical

Research Council Clinical and Population Cytogenetics Unit, and to the Nuffield Foundation and the Melville Trust for generous financial assistance. We would also like to thank Mrs Joan Beattie for expert care of our mice.

### REFERENCES

HEWITT, H. B., BLAKE, E. R. & WALDER, A. S. (1976) A Critique of the Evidence for Active Host Defence against Cancer, Based on Personal Studies of 27 Murine Tumours of Spontaneous Origin. *Br. J. Cancer*, **33**, 241.

MCKHANN, C. F. (1964) The Effect of X-ray on the Antigenicity of Donor Cells in Transplantation Immunity. *J. Immun.*, **92**, 811.

PETO, R., PIKE, M. C., ARMITAGE, P., BRESLOW, N. E., COX, D. R., HOWARD, S. V., MANTEL, N., MCPHERSON, K., PETO, J. & SMITH, P. G. (1977) Design and Analysis of Randomized Clinical Trials Requiring Prolonged Observation of Each Patient. II. Analysis and Examples. *Br. J. Cancer*, **35**, 1.

WOODRUFF, M. F. A. (1975) Tumor Inhibitory Properties of Anaerobic Corynebacteria. *Transplant. Proc.*, **7**, 229.

WOODRUFF, M. F. A. (1977*a*) Prospects for Immunotherapy. *Proc. Symp. Biological Preparations in the Treatment of Cancer*. Ed. A. H. Griffith and R. H. Regamey. Basel: S. Karger.

WOODRUFF, M. F. A. (1977*b*) Prospects for Immunotherapy of Solid Tumours. *Proc. 3rd International Congress of Immunology. Camberra: Aust. Acad. Sci.*, (In press).

WOODRUFF, M. F. A. & BOAK, J. L. (1966) Inhibitory Effect of Injection of *C. parvum* on the Growth of Tumour Transplants in Isogenic Hosts. *Br. J. Cancer*, **20**, 345.

WOODRUFF, M. F. A. & DUNBAR, N. (1973) The Effect of *Corynebacterium parvum* and Other Reticuloendothelial Stimulants on Transplanted Tumours in Mice. *Ciba Found. Symp., New Series* 18, *Immunopotentiation*. Ed. G. E. W. Wolstenholme and J. Knight. Amsterdam: ASP. p. 287.

WOODRUFF, M. F. A. & DUNBAR, N. (1975) Effect of Local Injection of *C. parvum* on the Growth of a Murine Fibrosarcoma. *Br. J. Cancer*, **32**, 34.

WOODRUFF, M. F. A., DUNBAR, N. & GHAFFAR, A. (1973) The Growth of Tumours in T-Cell Deprived Mice and Their Response to Treatment with *Corynebacterium parvum*. *Proc. R. Soc. Lond. B.*, **184**, 97.

WOODRUFF, M. F. A., GHAFFAR, A. & WHITEHEAD, V. L. (1976) Modification of the Effect of *C. parvum* on Macrophage Activity and Tumour Growth by X-irradiation. *Int. J. Cancer*, **17**, 652.

WOODRUFF, M. F. A. & WARNER, N. L. (1977) Effect of *Corynebacterium parvum* on Tumour Growth in Normal and Athymic (Nude) Mice. *J. natn. Cancer Inst.*, **58**, 111.

WOODRUFF, M. F. A. & WHITEHEAD, V. L. (1977) Mechanism of Inhibition of Immunization with Irradiated Tumour Cells by a Large Dose of *Corynebacterium parvum*. *Proc. R. Soc. Lond. B.*, **197**, 505.