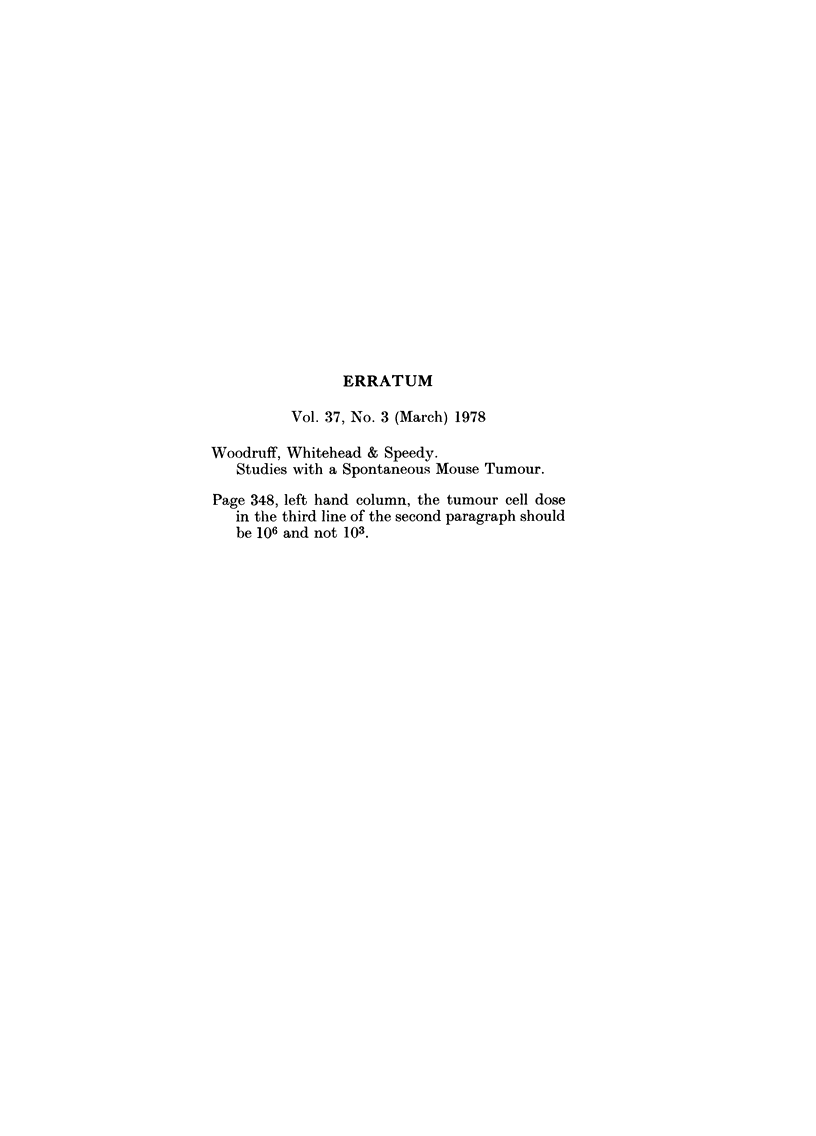# Erratum

**Published:** 1978-03

**Authors:** 


### ERRATUM

Vol. 37, No. 3 (March) 1978

Woodruff, Whitehead & Speedy.
Studies with a Spontaneous Mouse Tumour.

Page 348, left hand column, the tumour cell dose in the third line of the second paragraph should be $10^6$ and not $10^3$.